# Influence of respiratory mechanics and drive on genioglossus movement under ultrasound imaging

**DOI:** 10.1371/journal.pone.0195884

**Published:** 2018-04-16

**Authors:** Benjamin C. H. Kwan, Rachel A. McBain, Billy L. Luu, Jane E. Butler, Lynne E. Bilston, Simon C. Gandevia

**Affiliations:** 1 Neuroscience Research Australia (NeuRA), Barker St, Sydney, NSW, Australia; 2 Prince of Wales Clinical School, Faculty of Medicine, University of New South Wales, Sydney, NSW, Australia; University of Bari, ITALY

## Abstract

Genioglossus is the largest upper airway dilator and its dilatory movement can be measured non-invasively with magnetic resonance imaging and ultrasound. The present study used a novel ultrasound method to assess genioglossus movement in conditions in which ventilatory drive or respiratory mechanics were changed.

**Methods:**

Twenty healthy subjects (10 males, age 28±5 years [mean ± SD]) lay supine, awake, with the head in a neutral position. Ventilation was monitored with inductance bands. Real-time B-mode ultrasound movies were analysed. We measured genioglossus motion (i) during spontaneous breathing, voluntary targeted breathing (normal tidal volume Vt), and voluntary hyperpnoea (at 1.5Vt and 2 Vt); (ii) during inspiratory flow resistive loading; (iii) with changes in end-expiratory lung volume (EELV).

**Results:**

Average peak inspiratory displacement of the infero-posterior region of genioglossus was 0.89±0.56 mm; 1.02±0.88 mm; 1.27±0.70 mm respectively for voluntary Vt, and during voluntary hyperpnoea at 1.5Vt and 2Vt. A change in genioglossus motion was observed with increased Vt. During increasing inspiratory resistive loading, the genioglossus displaced less anteriorly (p = 0.005) but more inferiorly (p = 0.027). When lung volume was altered, no significant changes in genioglossus movement were observed (p = 0.115).

**Conclusion:**

In healthy subjects, we observed non-uniform heterogeneous inspiratory motion within the inferoposterior part of genioglossus during spontaneous quiet breathing with mean peak displacement between 0.5–2 mm, with more displacement in the posterior region than the anterior. This regional heterogeneity disappeared during voluntary targeted breathing. This may be due to different neural drive to genioglossus during voluntary breathing. During inspiratory resistive loading, the observed genioglossus motion may serve to maintain upper airway patency by balancing intraluminal negative pressure with positive pressure generated by upper airway dilatory muscles. In contrast, changes in EELV were not accompanied by major changes in genioglossus motion.

## Introduction

The human upper airway is important in many voluntary and involuntary tasks such as swallowing, speech and breathing. Patency of the upper airway requires a dynamic coordinated system that can rapidly change dilator muscle activity to counterbalance pressures that act to collapse it [1, 2]. The system varies with the respiratory cycle, with changes in head positions and posture, and with the state of wakefulness and sleep [3–9]. Passive mechanical properties of the upper airway and surrounding tissues determine its propensity to move and collapse [2, 10]. The system is also influenced by active moment-to-moment changes such as pressures generated as a result of neural drive to dilator and other upper airway muscles [10]. The largest upper airway dilator is genioglossus [11]. Neural drive to the human genioglossus increases in hypercapnic and hypoxic conditions [12–17], with a parallel increase to the hyoglossus [14]. Genioglossus electromyographic (EMG) activity has been reported to increase during inspiratory flow-resistive loading [18, 19]. Fogel and colleagues similarly found that phasic genioglossus EMG increases with increasing intrapharynegal negative pressure during passive ventilation, and inspiratory resistive loading [20]. During voluntary hyperventilation in the supine position, genioglossus EMG increases when compared to tidal breathing at rest [21, 22].

Genioglossus movement during quiet breathing has been measured with magnetic resonance imaging (MRI) [23–25] and ultrasound imaging [26]. Other imaging techniques have been used to examine the morphology and mechanical behaviour of the soft tissues in the upper airway, but are limited for quantifying genioglossus movement. MRI provides excellent upper airway and soft tissue resolution [27–29]. MR tagging studies revealed maximal anterior movement in the infero-posterior region of genioglossus of about 0.5–2 mm during tidal inspiration and posterior movement during expiration [23–25]. In healthy awake subjects, MRI recorded reduced overall anterior movement of genioglossus, axial plane cross-sectional airway area, and mid-sagittal plane anteroposterior airway diameter during inspiratory resistive loading [25, 30]. This is likely due to a shift in ability of upper airway dilator muscles to counteract the increased negative intraluminal pressure. Limitations of MRI include low temporal resolution, high cost, noise and limited availability.

Ultrasonography has been increasingly used to image the upper airway in real time in both experimental and clinical settings (e.g. [31–38]). More recently we reported a novel ultrasound research technique to measure genioglossus movement during quiet breathing in healthy subjects [26]. It has good consistency and reliability and showed genioglossus motion beginning before inspiratory airflow, with ~ 1 mm (predominantly anterior) displacement during inspiration. The technique revealed further heterogeneous (non-uniform) inspiratory motion within the postero-inferior genioglossus, with the posterior region displaced more than the anterior region.

Previous EMG studies confirmed increased genioglossus EMG in conditions with increased inspiratory neural drive, but quantitative measurement of regional genioglossus movement during inspiration in awake subjects is lacking. Therefore, to understand how altered respiratory mechanics and neural drive influence the physiological behaviour of genioglossus, the present study was designed to measure its movement using ultrasonography. We hypothesised that increased genioglossus inspiratory movement would occur in three selected physiological conditions in which inspiratory neural drive is increased: (i) during voluntary hyperpnoea, (ii) during inspiratory flow resistive loading, and (iii) with large changes in the end-expiratory lung volume (EELV), although anterior genioglossus movement would be counteracted by the forces produced by any changes in upper airway negative pressure.

## Materials and methods

Subjects with history of an active or chronic respiratory or sleep disorder and those using medication that could affect respiration or ventilation were excluded from the study. Thirty-eight healthy subjects (19 males and 19 females) in total were recruited across the three experiments. For each experiment we recorded data from twenty (10 males and 10 females) subjects. Some subjects participated in two or all three experiments. All participants completed the Epworth Sleepiness Scale [39, 40] and Berlin Sleep Questionnaire [41], two self-report questionnaires measuring subjective daytime sleepiness as predictive tools for obstructive sleep apnoea [42, 43]. Ethics approval was granted by the Human Research Ethics Committees of the Northern Sector of South East Sydney and Illawarra Area Health Service and University of New South Wales. The study was conducted according to the Declaration of Helsinki (2008) and informed written consent was obtained. The individual in this manuscript has given written informed consent (as outlined in PLOS consent form) to publish these case details.

### Experimental protocol

**Fig 1:**
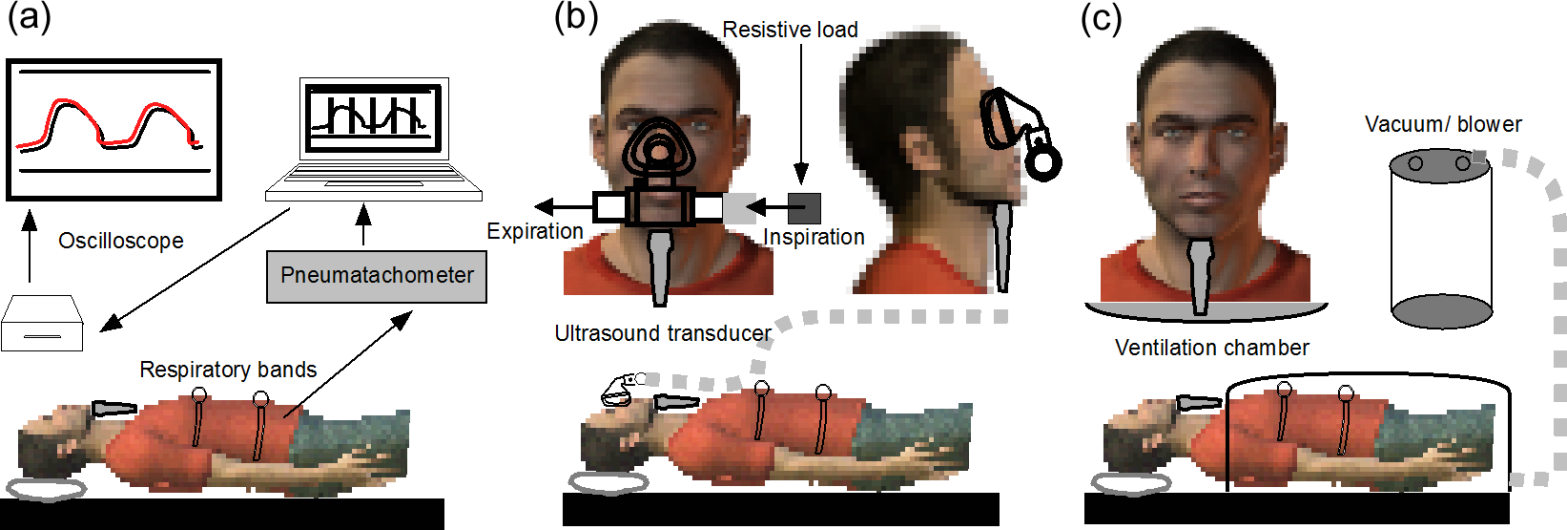
Setup for each of the 3 conditions. **Ultrasound transducer is positioned submentally.** (a) Voluntary hyperpnoea experiment. Subject lay supine and directly viewed the oscilloscope screen which displayed a signal of volume and a “target tracking” waveform based on the subject’s average tidal volume and respiratory frequency. The subject followed this waveform in real time. (b) Inspiratory resistive loading experiment. The subject wore a modified nose mask and were requested to breathe exclusively through the nose. The breathing apparatus was connected to a low resistive 2-way valve so that inspiration occurred through a pneumotachometer and pressure transducer whilst expiration took place at the valve to minimise rebreathing. The pressure, flow and respiratory inductance band signals were recorded in real time. An inspiratory resistive load was added by restriction of airflow distal to the pneumotachometer. (c) Imposed changes in end-expiratory level. The subject was supine in a head-out rigid-shell ventilation chamber. The chamber rested across the upper anterior chest just caudal to the suprasternal notch, with memory foam used to prevent pressure leak between chamber and torso. A vacuum/blower attachment was attached to the caudal part of the shell to allow changes in extra-thoracic pressure within the chamber.

The experimental setup is shown in Fig 1, and is similar to our previously published method [26]. Measurements were made with subjects in the supine posture. Head position was standardised with the Frankfort plane (defined by the inferior borders of the bony orbits and the upper margin of the auditory meatus) perpendicular to the horizontal bed surface to minimise variation in upper airway size due to flexion or extension of the head [44, 45]. The antero-posterior position of the head relative to the lower cervical spine was also standardised. The angle between horizontal plane and a line from tragus to spinous process of C7 vertebrae was constrained to 37–42°, and the angle between horizontal plane and a line from lateral angle of eye to tragus to 77–82°. Angle measurements were made with two goniometers, aligned along the measurement plane and the horizontal plane. Padding was used for the head, neck and shoulders to maintain the standardised posture. Head position was checked before capturing each image sequence to ensure no change in the measured angles from the start of the experiment. Subjects were asked to relax, remain awake, keep their mouth closed and to place the tongue in its usual ‘resting’ position, usually with its tip on the incisors. This tongue position has been described as close to the optimum genioglossus length for protrusion force [46, 47]. Subjects breathed through their nose throughout the study. The resting ventilation of each subject was monitored using calibrated respiratory inductance bands (Inductotrace, Ambulatory Monitoring Inc., Ardsley, New York, USA) over thorax and abdomen. Calibration was achieved with 800 mL bags. Real-time signals were digitised at 1 kHz using a CED1401 data acquisition system and Spike2 software (Cambridge Electronic Design, Cambridge, UK). Respiratory data were analyzed off-line to determine the inspiratory time, tidal volume and respiratory rate. The onset of inspiration was taken from the signal of the abdominal inductance band [26].

### Ultrasound scanning and analysis

**Fig 2:**
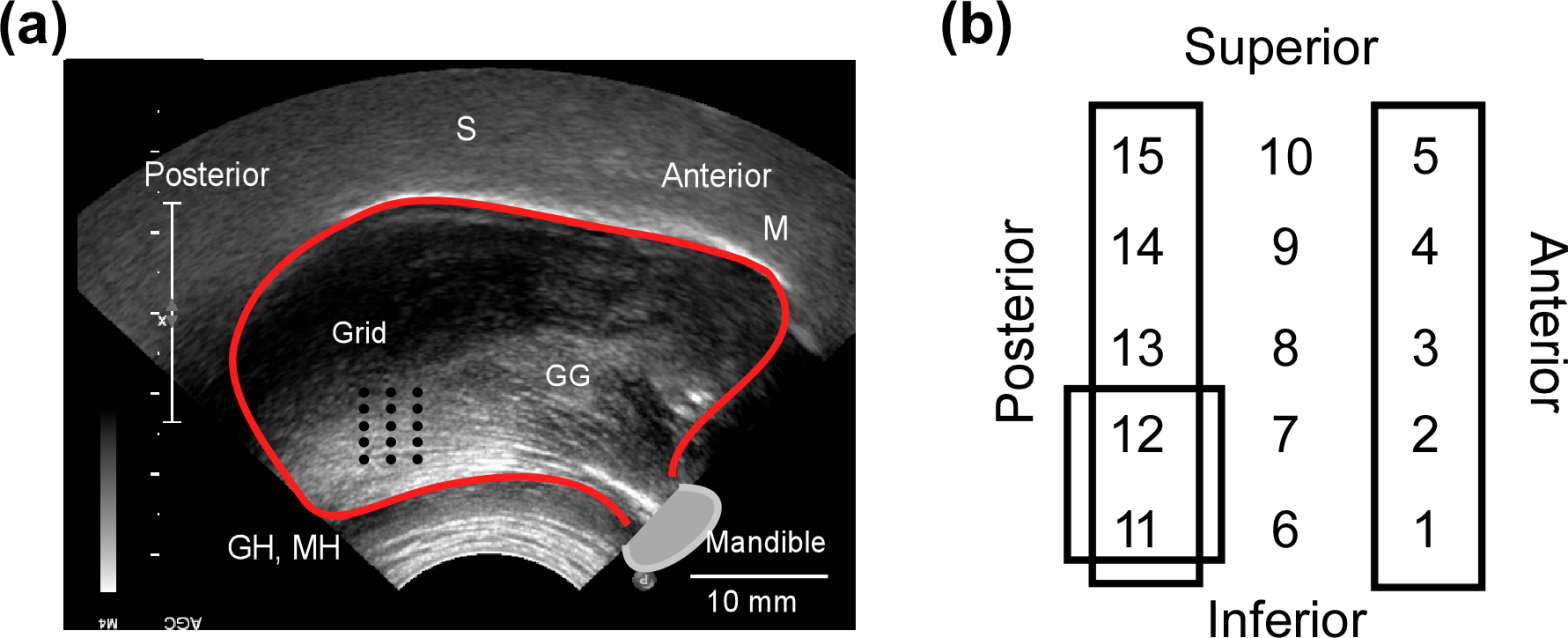
Schematic representation of ultrasound image, grid position and numbering system. (a) Ultrasound image of the placement of grid with red line outlining the genioglossus and 15 black dots denoting the tracking points. GG–genioglossus, M–mucosa, S–tongue surface, GH–geniohyoid, MH–myohyoid (b) 15 grid points and columns represented. Points 1–5 are defined as the “anterior” column, 6–10 as the “middle” column and 11–15 as the “posterior” column. Points 11, 12 are defined as the “infero-posterior” points.

Our ultrasound protocol has been shown to reliably image movement of the genioglossus during quiet breathing [26]. In brief, ultrasound images were collected using a Philips iU22 system (Andover, USA) with a curved array C8-5 transducer, which has a probe frequency of 5 to 8 MHz. The handheld transducer was positioned submentally, and aligned in the mid-sagittal plane and pointed cranially. This provided a lateral view of the tongue body, submental musculature and mandible. Time gain compensation, depth and near gain control were adjusted manually to obtain the best image quality (refer to Fig 2 for representative ultrasound image). The depth of the image acquisition was set to 6 cm. Output from the abdominal inductance band was recorded concurrently by the ultrasound machine, appearing as a waveform onscreen. This allows the synchronisation of genioglossus motion with respiration.

A scan of the surrounding tissues was also performed prior to recording to determine the position of the superior surface of the tongue, the mandible and the posterior portion of the tongue. Real-time B-mode images were collected for at least 5 consecutive stable breaths at a frame rate of ~40 Hz (termed a “sequence”). A minimum of three sequences were captured in each imaging session.

Image sequences were analyzed off-line using custom image correlation software developed in MATLAB (Mathworks, MA, USA). For regions of suitable image quality, a rectangular grid measuring around 100mm^2^ area containing 3 columns (≈ 4 mm apart) of 5 points (≈ 3 mm apart) was then placed over the tongue. Grid point 1 was positioned approximately 2 cm posterior to and 1 cm rostral to the internal spine of the mandible, where maximal inspiratory displacement was demonstrated in our previous study [26]. The nomenclature for the grid points is given in Fig 2. For analysis, points 1–5 are defined as the “anterior” column, 6–10 as the “middle” column and 11–15 as the “posterior” column. Points 11 and 12 are defined as the “infero-posterior” points.

We excluded any sequence in which swallowing or jaw motion occurred. We then selected the sequence with the clearest images. A custom image correlation program tracked the movement of these markers throughout the video sequence [48, 49]. In our published protocol[26], regional displacements over the three most stable breaths in each sequence were analyzed and the average resultant was calculated. Reported results in each experiment are the mean maximal inspiratory regional genioglossus displacement across 3 breaths for 20 subjects.

### Genioglossus motion during voluntary hyperpnoea

For each subject, the calibrated volume signal derived from the summed output of the two respiratory inductance bands was displayed on a digital oscilloscope (Tektronic TDS3014, Beaverton, Oregon, USA) directly above the subject within their field of view (Fig 1). Subjects first performed spontaneous quiet breathing without visual feedback. Three sequences during tidal breathing were then captured, termed “spontaneous tidal breathing”. The tidal volumes during these sequences were averaged. We then generated a “target” waveform based on average tidal volume and respiratory frequency. This was the “baseline” target-breathing pattern. A “tracking” waveform was then generated and the subject then practiced matching their real-time volume signal to the target waveform on screen. Once the lines were matched reasonably, 3 breathing sequences were captured, termed “voluntary tidal breathing” (Vt). Next, the participant tracked a signal that was 150% (1.5Vt) and 200% (2Vt) of the initial averaged tidal volume in random order, while maintaining the same breathing frequency. Subjects rested for 10 mins between each condition. During all tests, subjects were monitored to ensure they remained calm and alert.

### Genioglossus motion with inspiratory resistive loading

Subjects breathed through a tight-fitting nose mask. The mask was connected to a low-resistance, two-way valve, so that inspiration occurred through a pneumotachometer (model 3813, Hans Rudolph Inc, Kansas City, USA) and pressure transducer (DP45-16, Validyne Engineering Corp., Northbridge, USA) (Fig 1). The inspiratory line was connected to the pneumotachometer and pressure transducer via 50-mm and 3-mm diameter stiff tubes, respectively. Inspiratory resistive loads were applied by placement of 3 different calibrated cylindrical plastic blocks (30 mm long), each with a central hole of different diameter into the inspiratory line to restrict airflow distal to the pneumotachometer. These added an inspiratory resistance of 11.6 cmH_2_O/l/s, 22.3 cmH_2_O/l/s, and 75.2 cmH_2_O/l/s, loads A, B and C, respectively. Ultrasound recording of genioglossus movement was performed once steady-state breathing was achieved.

### Genioglossus motion with imposed changes in end-expiratory lung volume

Subjects rested supine in a custom-made head-out rigid-shell external ventilator chamber. The chamber was made of polymethyl methacrylate on a light reinforced stainless steel skeleton. Memory foam was used to prevent a pressure leak at the head end, and placed over the second and third ribs anteriorly. A vacuum/blower attachment was connected to the caudal part of the shell, to change extra-thoracic pressure and thus lung volume (Fig 1). Extra-thoracic pressure was measured inside the chamber via a pressure transducer (DP45-16, Validyne Engineering Corp., Northbridge, USA). Changes in end expiratory lung volume (EELV) were measured with the calibrated inductance bands (see above). Chamber pressure was adjusted to increase EELV by ~1 L or to decrease it by ~0.5 L from baseline, in random order. Lung volume changes were assessed by changes in the EELV indicated by the respiratory inductance bands. Ultrasound recording of genioglossus movement was performed once a stable end expiratory level was achieved.

Once analysis of this study was completed, the maximal resultant inspiratory displacement of the 15 grid points was less than in other experiments reported here (see Results) as well as our previous reports [26]. Therefore, a further study was performed with 6 subjects (3 males and 3 females) randomly selected from the original set of 20 subjects to determine if the presence of the ventilatory chamber across the anterior chest wall contributed to the reduced movement. Six sequences of tidal breathing were collected, half with the ventilator shell positioned over the chest and body in a random order. Ultrasound recording of genioglossus movement was performed in the usual way once stable breathing was achieved.

### Statistical analysis

Means and standard deviation (SD) were used for descriptive purposes. To assess gender differences in the subject characteristics we used independent sample t-tests. To assess differences in maximal displacement in different genioglossus regions across different conditions, a one-way ANOVA with repeated measures with a Greenhouse-Geisser correction was used. Two-way ANOVA analysis was used to compare the effect of gender on the magnitude of regional GG displacement. Post-hoc analyses were performed with Bonferroni’s correction, or Tukey’s test for pairwise comparison. Statistical analyses were carried out using SPSS version 23.0 (Armonk, NY, USA). Statistical significance was accepted at p<0.05.

## Results

Subject characteristics are shown in Table 1. In each experiment, there was no difference between the genders for age, Epworth sleepiness score and Berlin questionnaire, but females had a smaller neck circumference than males (31.8 ± 1.7 cm vs. 37.6 ± 2.4 cm; mean ± SD) [t(38) = 8.622, p<0.001, and lower BMI (21.0 ± 2.0 vs. 23.2 ± 2.9 kg/m^2^) [t(38) = 2.695, p = 0.011].

**Table 1:** Characteristics of the total pool of 38 subjects.

	Male (n = 19)	Female (n = 19)	p value
Age (years)	30.4 ± 4.9, (21–38)	27.6 ± 4.1, (21–37)	0.060
BMI (kg/m^2^)	23.2 ± 2.9, (17.3–27.4)	21.0 ± 2.0, (17.5–24.5)	0.011
Neck circumference (cm)	37.6 ± 2.4, (35–41)	31.8 ± 1.7, (28–34)	<0.001
Epworth sleepiness score (range 1–24)	3.1 ± 1.7, (1–7)	2.4 ± 1.6, (1–7)	0.250
Berlin questionnaire	Low	Low	

Data are expressed as mean ± SD with the range given in brackets. Body mass index (BMI). An Epworth sleepiness score of 11–24 is indicative of increased daytime sleepiness [40]. The Berlin questionnaire has 3 categories related to the risk of sleep apnoea: “high risk” if there are 2 or more categories where the score is positive, and “low risk” if there is only 1 or no categories where the score is positive [41].

The location and characteristics of the grid positioned over genioglossus, and subjects’ head positions are shown in Table 2 for each experimental condition (see Fig 2 and Methods for definition of grid points). Data in S1, S2 and S3 Tables reports the mean maximal inspiratory displacement of each of the 15 grid points during all 3 experiment conditions for 20 subjects.

**Table 2:** Image grid characteristics across experiments.

	Voluntary hyperpnoea	Lung volume alteration	Inspiratory resistive load
Mean posterior distance of point 1 from internal mental spine of the mandible (mm)	23.5 ± 4.4	17.7 ± 5.0	22.4 ± 5.0
Mean rostral distance of point 1 from internal mental spine of the mandible (mm)	13.2 ± 2.7	12.7 ± 3.5	11.8 ± 2.4
Mean distance between grid columns (mm)	3.3 ± 0.3	4.4 ± 0.5	3.3 ± 0.4
Mean distance between grid rows (mm)	2.5 ± 0.2	3.1 ± 0.4	2.3 ± 0.3
Mean angle α (°)	78.4 ± 2.9	78.4 ± 1.9	80.8 ± 1.8
Mean angle β (°)	38.8 ± 2.7	39.4 ± 2.1	40.5 ± 2.1

Data are expressed as mean ± SD. For image analysis, the internal mental spine of the mandible denotes the point of insertion of GG). Angle α denotes angle between the horizontal plane and a line from the tragus to the spinous process of the C7 vertebrae. Angle β denotes angle between the horizontal plane and a line from lateral angle of eye to the tragus.

### Voluntary hyperpnoea

For the 20 subjects, the mean respiratory rate was not significantly different between the 4 experimental conditions; spontaneous breathing, targeted voluntary to Vt, 1.5Vt, and 2Vt. The mean tidal volume increased close to the expected target of 50% and 100% above baseline spontaneous breathing tidal volume (Table 3). During spontaneous breathing, there was overt anterior movement of the genioglossus as reported previously [26]. On average (across all 15 points of the grid) the anterior movement was 0.88 ± 0.13 mm, and the overall resultant measured 1.03 ± 0.51 mm (Table 4 and S1 Table).

**Table 3:** Respiratory variables for the voluntary hyperpnoea experiment.

	Spontaneous tidal breathing	“Target” baseline tidal breathing	1.5x “Target” baseline	2x “Target” baseline	p value
Tidal volume (mL)	394 ± 161	419 ± 165	625 ± 265	752 ± 264	<0.001
Respiratory rate (breaths / min)	13.9 ± 4.7	13.9 ± 4.6	14.2 ± 5.0	14.1 ± 4.9	0.510
Respiratory cycle length (sec)	4.88 ± 1.96	4.86 ± 2.02	4.82 ± 2.10	4.84 ± 2.07	0.072
Inspiratory time (sec)	1.79 ± 0.90	2.01 ± 0.88	2.02 ± 0.90	2.06 ± 1.03	<0.001

Data are expressed as mean ± SD. There was no significant difference between the mean tidal volumes during spontaneous tidal breathing and “target” baseline tidal breathing experiments across 20 subjects (p = 0.06). There was no significant difference between the mean inspiratory time during all 3 “Target” breathing experiments across 20 subjects (p = 1.000)

**Table 4:** Average displacement of infero-posterior region of genioglossus (voluntary hyperpnoea).

	Mean across 15 points	Anterior column	Middle column	Posterior column	Infero-posterior points
Spontaneous tidal	1.03 ± 0.51	0.93 ± 0.49	1.00 ± 0.52	1.16 ± 0.56	1.13 ± 0.54
“Target” baseline tidal	0.83 ± 0.56	0.84 ± 0.56	0.83 ± 0.60	0.85 ± 0.55	0.89 ± 0.56
1.5 x “Target”	1.07 ± 0.89	1.08 ± 0.92	1.14 ± 0.95	1.00 ± 0.81	1.02 ± 0.88
2x “Target”	1.24 ± 0.59	1.24 ± 0.58	1.29 ± 0.60	1.21 ± 0.61	1.27 ± 0.70
p value	0.042	0.055	0.028	0.034	0.081

Mean maximal inspiratory displacement (mm) of different grid columns within genioglossus for 20 subjects. See Methods for grid column definition. Data are expressed as mean ± SD.

**Fig 3:**
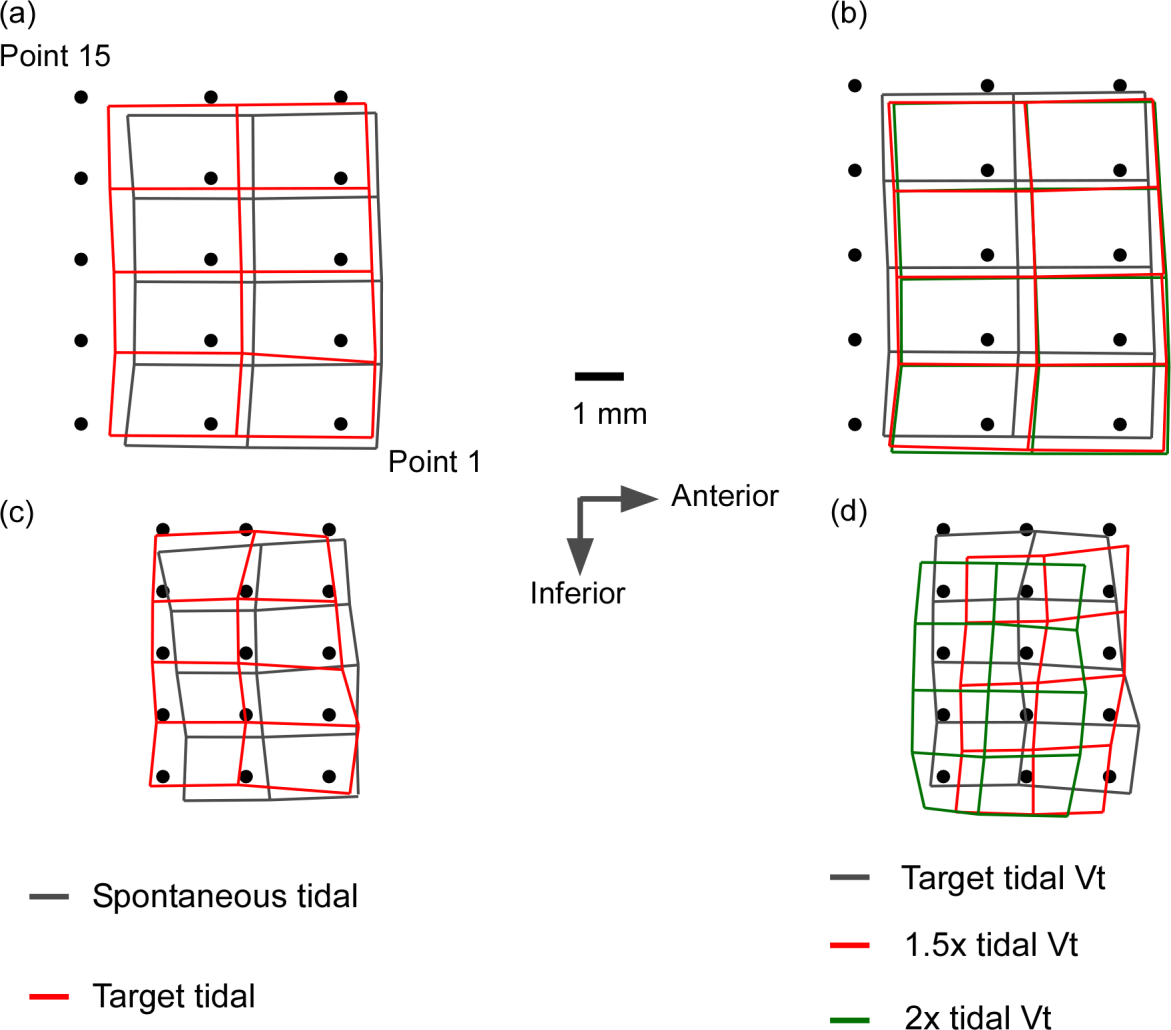
Mean peak inspiratory movement of grid during voluntary hyperpnoea experiment. Mean for the 15 grid points at the start of the respiratory cycle are denoted as solid circles. Fig 3A and 3C Spontaneous and targeted tidal breathing. Fig 3B and 3D During voluntary study with two volumes. Fig 3A and 3B Mean for 20 subjects. Fig 3C and 3D Result for one of the study subject.

During voluntary breathing, the mean resultant peak displacements of the inferoposterior region increased with increasing tidal volume to 0.89 ± 0.56 mm, 1.02 ± 0.88 mm and 1.27 ± 0.70 mm respectively for the “target tidal”, “1.5Vt” and “2Vt” conditions (Table 4, see Fig 3 for mean inspiratory movement of the grid). A significant difference was observed in the magnitude of peak resultant movement between the 3 “target breathing” conditions (ANOVA with repeated measures with a Greenhouse-Geisser correction, F_1.746,33.168_ 4.488, p = 0.023). There were non-significant changes in resultant displacement when subjects increased their targeted tidal volume by 1.5 times from “target tidal” baseline (1.02 ± 0.88 mm vs. 0.89 ± 0.56 mm, respectively, p = 0.483) or when targeted tidal volume increased from 1.5 times baseline Vt to 2 times baseline Vt (1.02 ± 0.88 mm vs. 1.27 ± 0.70 mm, respectively, p = 0.577). However, there was a statistically significant difference in the magnitude of the peak resultant displacement between “target tidal” and 2 times baseline Vt (p = 0.007). There was no significant interaction between gender and voluntary Vt on the maximal resultant displacement across the 15 grid point (two-way ANOVA F_(2,54)_ = 0.033, p = 0.967).

Further analysis demonstrated significant regional variation in the magnitude of the peak resultant movement within the focused infero-posterior part of genioglossus. During “spontaneous tidal” breathing, the posterior region moved 16% and 25% more than the middle or anterior regions respectively (F_1.275,24.22_16.484, p<0.001) (Table 5). Similar to our previous finding [26], maximal displacement was recorded for the most infero-posterior grid points (11,12) measuring 1.13 ± 0.54 mm. In contrast, during the 3 voluntary breathing conditions, this regional variation was lost, with all 3 regions moving in a more uniform “en-bloc” pattern (F_1.451,27.578_ 0.073, p = 0.874).

**Table 5:** Respiratory variables for the inspiratory resistive load experiment.

	Spontaneous tidal breathing	Load A	Load B	Load C	p value
Tidal volume (mL)	493 ± 169	510 ± 140	510 ± 157	605 ± 266	-
Respiratory rate (breaths / min)	13.3 ± 3.5	13.0 ± 3.9	12.8 ± 3.7	9.7 ± 4.0	<0.001
Respiratory cycle length (sec)	4.94 ± 1.84	5.20 ± 2.36	5.09 ± 1.51	7.23 ± 3.00	<0.001
Inspiratory time (sec)	1.30 ± 0.49	1.44 ± 0.35	1.49 ± 0.57	2.02 ± 1.42	0.093
Borg score	1.0 ± 1.0	2.5 ± 1.45	3.7 ± 1.73	6.0 ± 1.63	<0.001

Data are expressed as mean ± SD. Load A, B and C added an inspiratory resistance of 11.6 cmH_2_O/L/s, 22.3 cmH_2_O/L/s, and 75.2 cmH_2_O/L/s respectively. The mean respiratory rate was significantly lower only during inspiratory resistive loading (experiment C).

### Inspiratory resistive loading

When the 20 subjects inspired against an external inspiratory resistive load, inspiratory time and tidal volume increased with increasing resistance (Table 5).

**Fig 4:**
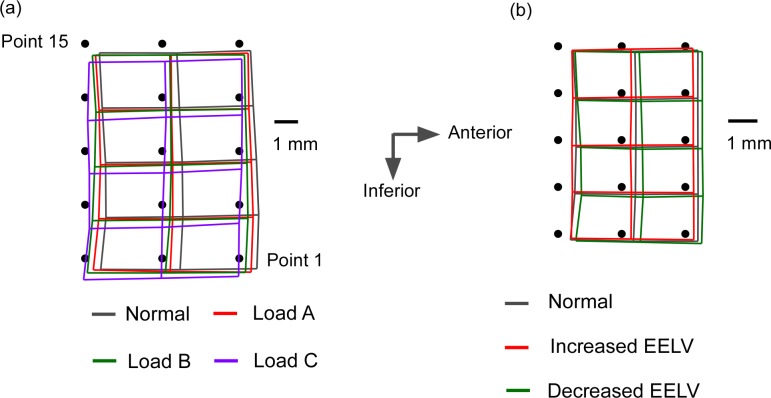
Mean peak inspiratory movement of grid for 20 subjects during resistive inspiratory load experiment and lung volume alteration experiment. Mean for the 15 grid points at the start of the respiratory cycle are denoted as solid circles. (a) During inspiratory resistive loading experiment. Load A, B and C added an inspiratory resistance of 11.6 cmH_2_O/L/s, 22.3 cmH_2_O/L/s, and 75.2 cmH_2_O/L/s respectively. (b) During passive change in EELV experiment.

Compared to spontaneous tidal breathing, tidal volume increased by around 23% when subjects inspired against the highest resistance of 75.2 cmH_2_O/L/s. During spontaneous breathing, anterior movement of the genioglossus again occurred. The mean anterior displacement of the grid across 20 subjects was 0.73 ± 0.12 mm and the mean resultant movement was 0.89 ± 0.43 mm. Table 6 shows the mean resultant across the 15 points, and for the anterior, middle and posterior grid columns within the infero-posterior part of genioglossus. Mean peak inspiratory movement of the grid during different inspiratory resistive loading is shown in Fig 4.

**Table 6:** Average displacement of infero-posterior region of genioglossus (inspiratory resistive load).

	Mean across 15 points	Anterior column	Middle column	Posterior column	Infero-posterior points
Spontaneous tidal	0.89 ± 0.43	0.82 ± 0.38	0.94 ± 0.48	0.92 ± 0.47	0.97 ± 0.54
Load A	0.78 ± 0.38	0.77 ± 0.45	0.78 ± 0.38	0.81 ± 0.40	0.79 ± 0.36
Load B	0.82 ± 0.55	0.77 ± 0.50	0.84 ± 0.59	0.89 ± 0.57	0.95 ± 0.55
Load C	1.18 ± 0.57	1.07 ± 0.52	1.24 ± 0.59	1.26 ± 0.64	1.32 ± 0.71
p value	0.043	0.102	0.030	0.031	0.024

Mean maximal inspiratory displacement (mm) of different grid columns within genioglossus for 20 subjects. See Methods for grid column definition. Data are expressed as mean ± SD. Load A, B and C added an inspiratory resistance of 11.6 cmH_2_O/L/s, 22.3 cmH_2_O/L/s, and 75.2 cmH_2_O/L/s respectively.

With increasing inspiratory resistive load, maximal anterior displacement of the grid points decreased progressively. This was statistically significant (F_1.623,30.841_ 7.017, p = 0.005). A Tukey post hoc test revealed this maximal anterior displacement was significantly less with load “A” (0.47 ± 0.40 mm, p = 0.009), load “B” (0.30 ± 0.59 mm, p<0.001) and load “C” (0.08 ± 0.81 mm, p = 0.02) compared to spontaneous breathing without a resistive load (0.73 ± 0.12 mm) (Tables 6 and S2). Maximal inferior displacement increased with increased inspiratory resistive load. It averaged 0.53 ± 0.33 mm, 0.57 ± 0.47, and 0.85 ± 0.62 mm along the coronal plane with load “A”, “B” and “C” respectively (Tables 6 and S2). The result was also statistically significant (F_2.211,42.005_ 3.777, p = 0.027). No gender influences on the magnitude of maximal inspiratory resultant displacements were observed [(F_(3,72)_ = 1.605, p = 0.196)].

We again recorded significant non-uniform inspiratory motion within the infero-posterior genioglossus, with the posterior region moving more than the anterior or middle region during spontaneous breathing (F_1.735,30.558_4.165, p = 0.029), with inspiratory resistive load “B” (F_1.99,34.401_4.651, p = 0.016), and with inspiratory resistive load “C” (F_1.976,37.538_10.748, p<0.0001), but not during inspiratory resistive load “A” (p = 0.64) (Table 6). Maximal displacement was recorded for the most infero-posterior grid point, measuring 0.97 ± 0.54 mm during tidal breathing.

### Change in lung volume produced by an external ventilator

During spontaneous breathing at a normal EELV with subjects lying supine in an external ventilator chamber, overt anterior GG movement was again observed. The mean anterior movement for the 20 participants across all grid points was 0.35 ± 0.11 mm, with a mean resultant displacement of 0.39 ± 0.21 mm (Tables 7 and S3 for 20 subjects and also for comparable displacements for 6 subjects inside and outside the ventilator chamber). Mean peak inspiratory movement of the grid during lung volume alteration is shown in Fig 4.

**Table 7:** Average displacement of genioglossus (lung volume alteration).

	Mean across 15 points	Anterior column	Middle column	Posterior column	Infero-posterior points
Spontaneous tidal	0.39 ± 0.21	0.32 ± 0.16	0.41 ± 0.22	0.52 ± 0.28	0.62 ± 0.37
Negative extra-thoracic pressure	0.33 ± 0.25	0.27 ± 0.31	0.31 ± 0.20	0.43 ± 0.30	0.49 ± 0.33
Positive extra-thoracic pressure	0.48 ± 0.29	0.40 ± 0.23	0.48 ± 0.31	0.57 ± 0.38	0.73 ± 0.46
p value	0.115	0.169	0.066	0.263	0.064
6 subjects outside chamber	0.81 ± 0.68	0.83 ± 0.71	0.86 ± 0.66	0.86 ± 0.62	0.97 ± 0.69
6 subjects inside chamber	0.49 ± 0.58	0.56 ± 0.61	0.56 ± 0.54	0.58 ± 0.52	0.62 ± 0.53
p value	0.011	0.038	0.037	0.029	0.033

Mean maximal inspiratory displacement (mm) of different grid columns within genioglossus for 20 subjects. See Methods for grid column definition. Data are expressed as mean ± SD.

When positive extrathoracic pressure was applied (15.7 ± 4.2 cmH_2_O), the end expiratory lung volume decreased by 532 ± 159 mL, with displacement of the measured grid points of 0.48 ± 0.29 mm during inspiration. When negative extrathoracic pressure was applied (16.6 ± 4.1 cmH_2_O), the end expiratory lung volume increased by 1067 ± 191 mL, and the average maximal displacement across all 15 grid points measured 0.33 ± 0.25 mm (Table 7 and Fig 4). The displacements were not statistically significantly different (F_1.486,28.243_2.474, p = 0.115). There was no significant gender influence on the magnitude of displacement [F_(2,54)_ = 0.185, p = 0.831].

Significant regional variation in the magnitude of the peak resultant motion during inspiration was again recorded, with the posterior region of the grid displaced more during spontaneous breathing (F_1.128,21.433_13.503, p = 0.001), decreased EELV (F_1.509,28.667_6.125, p = 0.01), and increased EELV (F_1.939,36.842_8.048, p = 0.001) (Table 7). Maximal displacement was again recorded for the most infero-posterior point, measuring 0.62 ± 0.37 mm.

In the further study with 6 subjects (3 males and 3 females) to determine if the presence of the ventilatory chamber across the anterior chest wall contributed to the reduced movement, the respiratory parameters were similar when subjects were inside or outside the ventilator chamber (S4 Table).

**Fig 5:**
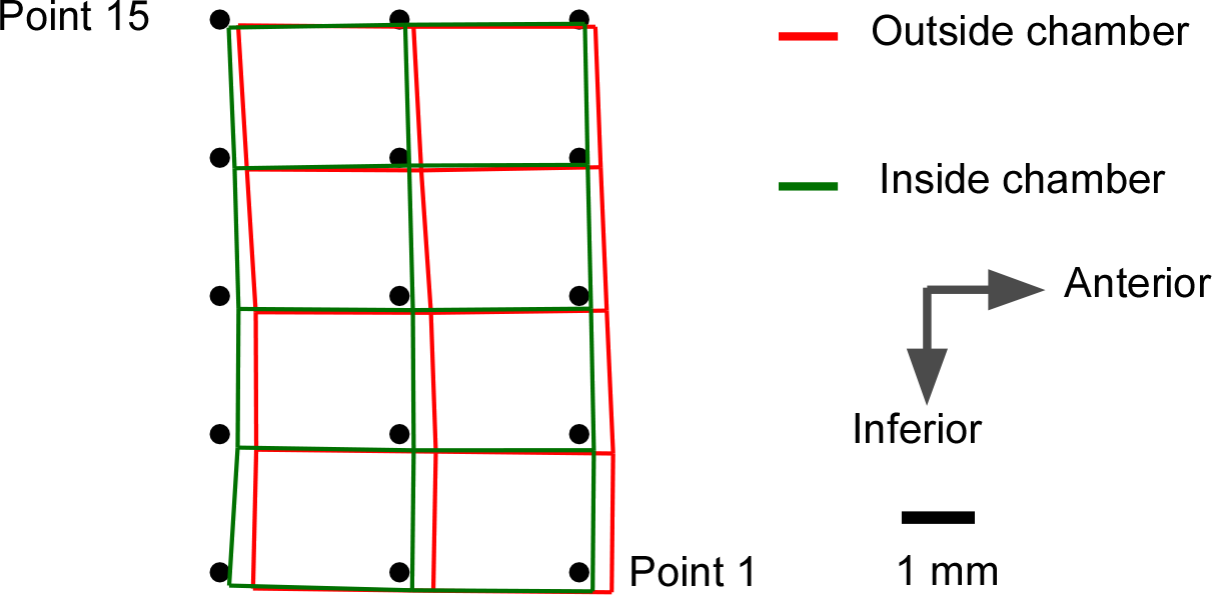
Mean peak inspiratory movement of grid for 6 subjects inside or outside chamber during lung volume alteration experiment. Mean for the 15 grid points at the start of the respiratory cycle are denoted as solid circles.

Maximal inspiratory displacement of all regions within the inferoposterior genioglossus significantly decreased when subjects were inside the ventilator chamber (Fig 5, lower part of Tables 7 and S3). The mean resultant peak displacement of the grid points was 0.81 ± 0.68 mm when the 6 subjects were outside the chamber and 0.49 ± 0.58 mm when subjects were inside the chamber (F_1,5_15.579, p = 0.011).

## Discussion

Using our recently developed ultrasound technique, this study reports how much genioglossus displacement changed when ventilatory drive was altered. When subjects voluntarily adopted a higher tidal volume during the voluntary hyperpnoea conditions, genioglossus displacement increased. With inspiration against an increasing resistive load, tidal volume and inspiratory time increased, and the genioglossus displaced less anteriorly, but more inferiorly. When lung volume was altered with the external ventilator chamber, no significant changes in genioglossus movement were observed. In the substudy however, genioglossus displacement was significantly decreased when the six subjects were inside the chamber, suggesting an influence from the occlusive foam placed across the upper anterior chest wall. Similar to our previous MRI and ultrasound study, we recorded non-uniform inspiratory motion within the inferoposterior part of genioglossus during quiet breathing with mean peak displacement between 0.5–2 mm, and more displacement in the posterior region than the anterior [24, 26]. However, this usual regional difference in motion was lost during voluntary targeted breathing, instead a more uniform anteroinferior “en bloc” motion was observed. This suggests a potential role for cortical inputs during voluntary breathing, resulting in increased displacement in the anterior part of genioglossus. Table 8 summarises our ultrasound findings and previous reported MRI and genioglossus EMG findings.

**Table 8:** Summary of genioglossus activity during different respiratory states.

	Movement (Ultrasound)	Movement (MRI)[24, 25]	Genioglossus EMG
Spontaneous tidal breathing	≈ 1 mm (anterior)	0.5–2 mm (anterior)	Variable EMG dependent of unit (tonic activation)
Voluntary hyperpnoea	↑ Vt ↑ ant mov’t	-	↑ Vt ↑ EMG activity
Inspiratory resistive load	↑ resistance ↑ ant mov’t	↑ resistance ↑ ant mov’t	↑ resistance ↑ EMG activity
Altered EELV	↓ EELV ↑ ant mov’t	-	↓ EELV↑ EMG activity

EMG–electromyography, Vt—tidal volume, mov’t–movement, EELV–end expiratory lung volume

With ultrasonography, we recorded a decrease in regional genioglossus anterior displacement and an increase in inferior displacement when the subjects inspired against a resistive load. This is similar to previous MRI studies observing more lateral narrowing of the upper airway and less anterior tongue motion during loaded inspiration [25, 30]. In previous studies of genioglossus EMG, adding inspiratory resistance resulted in increased inspiratory time, tidal volume and decreased airflow [50–52]. Inspiratory resistive loading likely caused greater negative epiglottic pressure [53–56], resulting in increased ventilatory drive to compensate with activation of upper airway dilator muscles and increased genioglossus EMG. These effects have been shown in wakefulness and sleep [50–52]. This increased negative upper airway pressure appears to be a major driver of genioglossus activation. This is supported by findings that despite the increased intrapharyngeal pressure during resistive loading, the increase in pharyngeal resistance is only modest [18, 57]. One hypothesis to explain the observed genioglossus motion is that moment to moment genioglossus activity serves to maintain upper airway patency, with overall motion determined by the balance of intraluminal negative pressure that acts to collapse it, and positive pressure generated by the upper airway dilatory muscles to open it [7, 58]. The biomechanical effect of this dynamic motion would ensure airway patency is maintained within and between breaths [2].

During voluntary hyperpnoea genioglossus displacement increased during inspiration. We also observed a more homogeneous “en bloc” anteroinferior movement during voluntary breathing, as compared to a heterogeneous pattern within the inferoposterior part of genioglossus during spontaneous breaths. This is consistent with previous EMG studies that recorded higher genioglossus EMG in the posterior compared to anterior region in quiet breathing [21]; greater genioglossus EMG without a significant difference between the most anterior and most posterior genioglossus regions during voluntary hyperpnoea or voluntary tasks when compared to quiet breathing; as well as greater differences in the phasic and tonic EMG components during voluntary tasks [21, 22]. The observed pattern of genioglossus motion may be the combined results of regional differences in muscle fiber type and composition between anterior and posterior genioglossus [59–61], different and additional inputs from the motor cortex, and inputs from the pontine and parabrachial nuclei to the hypoglossal motor nucleus during voluntary tasks [62–65].

Interestingly, when lung volume was altered by the external ventilator, a reduction or increase in end expiratory lung volume did not significantly change genioglossus displacement. This is in contrast to previous studies in which volitional or passive lowering of lung volumes during wakefulness occurred, whose findings include a reduction in upper airway size [66, 67], increased genioglossus intramuscular EMG [68], and changes in tracheal traction and pharyngeal collapsibility [69–72]. Furthermore, we found a decrease in regional genioglossus displacement when six subjects were inside the external ventilator. One possible explanation may be the activation of chest wall mechanoreceptors receiving cutaneous and proprioceptive inputs by application of foam across the upper anterior chest wall to ensure adequate seal of the ventilation chamber [73, 74].

In all our experiments where ventilatory drive was modulated, there were no significant differences between male and female subjects in the magnitude of maximal inspiratory infero-posterior regional genioglossus displacement. This is consistent with previous studies during wakefulness, although a significant increase in pharyngeal resistance (with or without inspiratory respiratory load) was observed in men during NREM sleep [75, 76]. In those studies, no gender differences were observed in central drive or response to loading, although greater genioglossus EMG was reported in awake women compared to men in one study [77]. Previous imaging studies have not observed significant sex-related differences in pharyngeal anatomy or pharyngeal dilator muscle activation during wakefulness in healthy subjects [78–81], or those with obstructive sleep apnoea [82, 83].

Our study has limitations. First, the actions of the intrinsic and/or extrinsic tongue muscles may have contributed to the motion within the chosen inferoposterior part of genioglossus. In rodents, minimal intrinsic muscle activity is recorded during eupnoea [84] but co-activation of intrinsic and extrinsic muscles can occur when drive is increased, as in hypoxia [85]. However, the region on which we focused shows the most motion during respiration [24, 26]. Second, epiglottic pressure, hypothesised to powerfully modulate central drive to genioglossus, was not measured in this study. However, our results are largely in keeping with previous MRI studies and genioglossus EMG studies, in which increased displacement occurs when ventilatory drive increases. Third, ultrasonography has intrinsic limitations. Detailed differentiation of the intramuscular architecture of genioglossus is difficult due to likely similar acoustic impedances within the muscle, and the image degradation produced by subject and transducer movements. However, we have previously demonstrated high intra-session and inter-session reliability with our method. Fourth, a variation in head and jaw position can influence the magnitude of measured posterior tongue motion during inspiration. In a recent MRI study, the largest motion was recorded with head in a neutral position standardised with the Frankfort plane and jaw open, and smallest with head extension. There was no significant difference between neutral and a mean flexion angle of 23°, but there is a reduction of ≈ 0.5mm in measured motion at a mean extension angle of 23° from neutral position [45]. In our study, the variation in head position is much smaller and likely to have minimal effect on measured motion.

## Conclusion

Our findings provide improved understanding and quantification of genioglossus movement during conditions where ventilatory drive is altered in healthy subjects. Increased central drive did not always result in increased genioglossus displacement. The magnitude and direction of the inferoposterior regional genioglossus displacement is a result of the balance between the negative intraluminal pressure and the positive dilatatory pressure provided by the upper airway muscles in order to maintain upper airway patency. We have demonstrated the ability of ultrasound to record genioglossus movement in a research setting, with findings similar to previous MRI studies [24, 25]. Furthermore, ultrasonography can perform this in real time, at lower cost and greater accessibility, allow rapid assessment of the biomechanical effect of genioglossus activation, and allow comparison to previous EMG studies, thus providing more understanding of the complex dynamic system required to ensure upper airway patency.

## Supporting information

S1 Table Mean maximal inspiratory displacement of 15 grid points during voluntary hyperpnoea experiment for 20 subjects. (PDF)

S2 Table Mean maximal inspiratory displacement of 15 grid points during inspiratory resistive load experiment for 20 subjects. (PDF)

S3 Table Mean maximal inspiratory displacement of 15 grid points during lung volume alteration experiment for 20 subjects. (PDF)

S4 Table Respiratory variables for the lung volume alteration experiment. (PDF)
