# Supplementary material for: Influence of respiratory mechanics and drive on genioglossus movement under ultrasound imaging
Source: PLoS One. 2018 Apr 16;13(4):e0195884. doi: 10.1371/journal.pone.0195884 (PMC5901985; doi:10.1371/journal.pone.0195884)
Supplement: S1 Table — (PDF) [file pone.0195884.s001.pdf]

Supporting Table 1. Mean maximal inspiratory displacement of 15 grid points during voluntary hyperpnoea experiment for 20 subjects.

	Point	1	2	3	4	5	6	7	8	9	10	11	12	13	14	15
**Spontaneous tidal breathing**	**A**	0.49	0.51	0.50	0.52	0.55	0.60	0.62	0.58	0.54	0.60	0.93	0.95	0.91	0.72	0.62
	**B**	0.47	0.48	0.44	0.36	0.36	0.58	0.49	0.45	0.39	0.36	0.54	0.46	0.42	0.37	0.37
	**C**	0.21	0.18	0.23	0.23	0.19	0.21	0.13	0.21	0.22	0.24	0.15	0.28	0.28	0.32	0.28
	**D**	0.30	0.25	0.22	0.27	0.21	0.22	0.34	0.33	0.34	0.35	0.19	0.38	0.33	0.33	0.36
	**E**	0.24	0.31	0.31	0.30	0.31	0.29	0.33	0.32	0.31	0.28	0.29	0.39	0.33	0.30	0.24
	**F**	0.76	0.78	0.63	0.52	0.49	0.98	0.94	0.74	0.60	0.57	1.15	1.28	1.00	0.97	0.85
	**G**	0.69	0.73	0.62	0.54	0.36	0.59	0.66	0.62	0.45	0.35	0.83	0.69	0.67	0.55	0.43
	**H**	0.64	1.29	1.34	1.31	1.22	0.61	1.21	1.44	1.28	1.25	0.66	1.13	1.54	1.65	1.38
	**I**	1.23	1.27	1.22	1.13	1.03	1.11	1.36	1.37	1.22	1.09	1.19	1.43	1.51	1.52	1.38
	**J**	0.20	0.19	0.15	0.13	0.14	0.18	0.21	0.18	0.19	0.16	0.21	0.30	0.28	0.27	0.26
	**K**	0.54	0.53	0.49	0.38	0.29	0.53	0.44	0.36	0.33	0.34	0.50	0.50	0.38	0.34	0.36
	**L**	0.72	0.72	0.70	0.67	0.71	0.78	0.82	0.81	0.88	0.88	0.92	1.01	1.00	0.96	0.73
	**M**	0.34	0.61	0.62	0.46	0.43	0.51	0.67	0.77	0.85	0.75	0.57	0.83	0.98	1.02	0.90
	**N**	0.37	0.29	0.26	0.17	0.17	0.30	0.15	0.16	0.23	0.24	0.37	0.44	0.50	0.47	0.42
	**O**	0.40	0.44	0.44	0.46	0.45	0.42	0.49	0.49	0.49	0.52	0.62	0.67	0.68	0.61	0.53
	**P**	0.87	0.89	0.84	0.83	0.76	0.98	1.00	0.98	0.99	1.01	1.05	1.10	1.13	1.14	1.25
	**Q**	0.92	0.88	0.99	1.00	1.04	0.82	0.95	1.09	1.12	1.18	1.01	1.14	1.02	0.99	0.85
	**R**	0.92	0.95	0.95	0.88	0.78	0.86	0.89	0.92	0.84	0.81	0.67	0.86	0.86	0.77	0.67
	**S**	0.44	0.50	0.59	0.57	0.50	0.38	0.54	0.62	0.64	0.64	0.29	0.53	0.65	0.70	0.79
	**T**	0.71	0.71	0.71	0.75	0.69	0.58	0.68	0.72	0.82	0.75	0.71	0.85	0.96	0.97	0.92
	**Mean ± SD**	**0.92 ± 0.45**	**1.01 ± 0.52**	**0.99 ± 0.53**	**0.93 ± 0.52**	**0.86 ± 0.50**	**0.93 ± 0.44**	**1.04 ± 0.55**	**1.06 ± 0.60**	**1.03 ± 0.56**	**1.00 ± 0.54**	**1.04 ± 0.53**	**1.23 ± 0.56**	**1.25 ± 0.62**	**1.21 ± 0.65**	**1.10 ± 0.58**
**"Target" tidal breathing**	**A**	0.70	0.77	0.74	0.81	0.69	0.26	0.52	0.58	0.55	0.58	0.46	0.27	0.33	0.29	0.40
	**B**	0.20	0.21	0.22	0.24	0.20	0.19	0.19	0.19	0.17	0.13	0.26	0.27	0.27	0.21	0.17
	**C**	0.76	0.76	0.61	0.46	0.27	0.44	0.64	0.71	0.60	0.43	0.21	0.40	0.36	0.35	0.36
	**D**	0.03	0.04	0.02	0.06	0.08	0.09	0.10	0.10	0.13	0.17	0.10	0.11	0.07	0.04	0.03
	**E**	0.09	0.13	0.13	0.22	0.22	0.12	0.16	0.16	0.14	0.10	0.17	0.22	0.13	0.16	0.17
	**F**	0.22	0.24	0.28	0.28	0.27	0.27	0.27	0.31	0.29	0.31	0.36	0.36	0.42	0.36	0.38
	**G**	0.98	0.91	0.80	0.63	0.40	1.04	0.97	0.78	0.58	0.35	0.89	0.82	0.73	0.55	0.39
	**H**	0.49	0.89	0.71	0.58	0.49	0.63	1.04	1.07	0.88	0.66	0.72	1.04	1.07	0.95	0.73
	**I**	1.01	0.81	0.79	0.73	0.80	1.02	1.00	0.90	0.93	0.93	0.93	1.22	1.25	1.20	1.21
	**J**	0.56	0.51	0.60	0.61	0.57	0.58	0.56	0.55	0.54	0.57	0.53	0.52	0.47	0.47	0.34
	**K**	0.42	0.50	0.33	0.19	0.12	0.18	0.12	0.20	0.18	0.14	0.25	0.16	0.22	0.23	0.15
	**L**	0.93	0.82	0.72	0.70	0.59	0.93	0.82	0.73	0.67	0.59	0.85	0.90	0.84	0.72	0.57
	**M**	0.29	0.30	0.17	0.13	0.11	0.17	0.13	0.17	0.29	0.29	0.38	0.44	0.40	0.49	0.50
	**N**	0.29	0.24	0.19	0.16	0.19	0.08	0.06	0.03	0.00	0.03	0.37	0.42	0.38	0.39	0.32
	**O**	0.35	0.35	0.42	0.38	0.34	0.42	0.36	0.42	0.31	0.21	0.33	0.30	0.22	0.15	0.21
	**P**	0.54	0.53	0.47	0.40	0.38	0.55	0.50	0.45	0.39	0.39	0.64	0.58	0.54	0.48	0.39
	**Q**	1.01	0.98	0.90	0.75	0.76	1.02	1.09	1.00	0.80	0.77	1.14	1.24	0.98	0.60	0.39
	**R**	0.14	0.15	0.22	0.24	0.19	0.21	0.20	0.22	0.19	0.16	0.20	0.22	0.27	0.29	0.31
	**S**	0.92	1.22	1.38	1.41	1.31	0.72	1.11	1.23	1.26	1.37	0.51	0.89	1.00	1.12	1.19
	**T**	1.11	1.27	1.31	1.21	1.08	1.12	1.28	1.33	1.27	1.16	1.08	1.28	1.28	1.22	1.19
	**Mean ± SD**	**0.89 ± 0.57**	**0.94 ± 0.60**	**0.89 ± 0.61**	**0.82 ± 0.58**	**0.73 ± 0.54**	**0.81 ± 0.58**	**0.90 ± 0.66**	**0.90 ± 0.64**	**0.82 ± 0.60**	**0.76 ± 0.59**	**0.84 ± 0.51**	**0.94 ± 0.62**	**0.91 ± 0.61**	**0.83 ± 0.58**	**0.76 ± 0.56**
**1.5 x "target" tidal**	**A**	0.68	0.76	0.54	0.35	0.24	0.66	1.01	1.03	0.92	0.58	0.21	0.28	0.35	0.41	0.34
	**B**	0.16	0.18	0.21	0.22	0.24	0.14	0.16	0.19	0.18	0.19	0.10	0.11	0.08	0.13	0.18
	**C**	1.03	0.84	0.70	0.56	0.39	0.54	0.72	0.83	0.73	0.55	0.56	0.80	1.05	1.12	0.83
	**D**	0.10	0.10	0.11	0.12	0.10	0.12	0.05	0.09	0.13	0.11	0.06	0.07	0.07	0.09	0.08
	**E**	0.35	0.31	0.24	0.25	0.27	0.30	0.31	0.27	0.26	0.27	0.33	0.29	0.49	0.43	0.28
	**F**	0.67	0.80	0.78	0.70	0.63	0.53	0.89	0.80	0.63	0.62	0.33	0.75	0.71	0.70	0.69
	**G**	0.65	0.49	0.27	0.07	0.10	0.55	0.45	0.29	0.25	0.10	0.54	0.45	0.37	0.28	0.20
	**H**	1.94	2.01	1.80	1.61	1.11	1.88	2.23	1.96	1.79	1.17	1.57	2.21	1.96	1.70	1.33

	**I**	1.26	1.18	1.09	0.89	0.68	1.00	1.14	1.05	0.97	0.92	0.59	0.93	0.97	0.96	0.90
	**J**	0.51	0.39	0.38	0.38	0.30	0.47	0.39	0.31	0.26	0.37	0.32	0.35	0.32	0.35	0.30
	**K**	0.57	0.48	0.42	0.40	0.39	0.61	0.56	0.50	0.57	0.49	0.59	0.64	0.58	0.61	0.57
	**L**	0.57	0.51	0.42	0.25	0.29	0.57	0.56	0.46	0.47	0.42	0.43	0.53	0.61	0.49	0.35
	**M**	0.52	0.62	0.56	0.53	0.40	0.60	0.64	0.61	0.54	0.43	0.66	0.70	0.68	0.62	0.53
	**N**	0.48	0.59	0.54	0.49	0.45	0.44	0.63	0.68	0.71	0.56	0.44	0.51	0.49	0.34	0.20
	**O**	0.22	0.22	0.25	0.36	0.32	0.24	0.31	0.29	0.26	0.28	0.21	0.22	0.23	0.23	0.28
	**P**	0.23	0.49	0.57	0.60	0.57	0.30	0.48	0.57	0.64	0.52	0.27	0.38	0.44	0.42	0.37
	**Q**	2.61	2.66	2.60	2.46	2.29	2.70	2.79	2.68	2.49	2.47	2.22	2.33	2.02	1.94	1.78
	**R**	0.67	0.45	0.38	0.31	0.30	0.76	0.60	0.51	0.38	0.32	0.66	0.60	0.53	0.43	0.30
	**S**	0.92	1.04	1.11	0.95	0.87	0.80	0.98	0.92	0.89	0.92	0.46	0.77	0.81	0.82	0.83
	**T**	1.02	1.02	0.91	0.94	0.91	0.98	1.03	0.92	0.91	0.87	0.89	0.90	0.82	0.80	0.72
	**Mean ± SD**	**1.22 ± 0.99**	**1.22 ± 1.00**	**1.12 ± 0.97**	**1.00 ± 0.91**	**0.87 ± 0.79**	**1.15 ± 0.98**	**1.29 ± 1.07**	**1.21 ± 1.00**	**1.13 ± 0.92**	**0.98 ± 0.84**	**0.92 ± 0.82**	**1.12 ± 0.96**	**1.09 ± 0.84**	**1.04 ± 0.78**	**0.89 ± 0.69**
**2 x "target" tidal breathing Vt**	**A**	0.77	0.83	0.80	0.77	0.74	0.59	0.78	0.80	0.73	0.57	0.40	0.51	0.60	0.70	0.64
	**B**	0.62	0.71	0.69	0.58	0.47	0.53	0.73	0.77	0.65	0.66	0.61	0.79	0.84	0.65	0.45
	**C**	1.33	1.72	1.65	1.14	0.84	0.89	1.35	1.31	1.27	1.19	0.73	0.95	1.17	1.24	0.84
	**D**	0.56	0.51	0.54	0.62	0.52	0.57	0.50	0.31	0.33	0.32	0.36	0.13	0.20	0.31	0.33
	**E**	0.73	0.66	0.62	0.59	0.54	0.66	0.60	0.54	0.47	0.43	0.52	0.51	0.33	0.21	0.21
	**F**	1.18	1.20	1.09	1.38	1.16	1.31	1.47	1.30	1.31	1.27	0.93	1.38	1.30	1.07	1.21
	**G**	0.53	0.49	0.43	0.29	0.30	0.51	0.47	0.45	0.33	0.17	0.55	0.49	0.49	0.36	0.22
	**H**	1.86	1.54	1.31	1.08	1.01	1.91	1.68	1.46	1.24	0.99	1.65	1.50	1.21	1.01	0.91
	**I**	1.32	1.05	0.93	0.96	0.87	1.42	1.24	1.08	1.00	0.88	1.53	1.37	1.29	1.19	1.02
	**J**	0.27	0.27	0.25	0.22	0.17	0.31	0.30	0.25	0.27	0.31	0.32	0.30	0.26	0.28	0.22
	**K**	0.91	0.78	0.72	0.80	0.68	0.96	0.94	0.85	0.73	0.71	0.57	0.77	0.75	0.67	0.62
	**L**	1.00	0.84	0.68	0.57	0.54	0.91	0.83	0.68	0.64	0.61	0.81	0.93	0.87	0.76	0.59
	**M**	0.46	0.56	0.58	0.50	0.40	0.45	0.56	0.64	0.56	0.55	0.26	0.45	0.58	0.56	0.58
	**N**	0.54	0.67	0.47	0.30	0.17	0.51	0.70	0.64	0.49	0.30	0.40	0.64	0.62	0.54	0.38
	**O**	0.21	0.14	0.15	0.14	0.12	0.17	0.20	0.23	0.21	0.24	0.13	0.18	0.30	0.40	0.45
	**P**	0.46	0.58	0.56	0.53	0.57	0.46	0.65	0.70	0.77	0.72	0.51	0.67	0.74	0.76	0.65
	**Q**	1.50	1.30	1.21	0.99	0.84	1.50	1.39	1.28	1.08	0.93	1.36	1.72	1.56	1.30	1.50
	**R**	1.22	0.91	0.80	0.61	0.42	1.36	1.12	0.89	0.63	0.35	1.04	0.97	0.76	0.54	0.32
	**S**	1.11	1.08	0.96	0.90	0.81	1.21	1.11	1.01	0.96	0.87	1.22	1.10	0.99	0.98	0.90
	**T**	1.36	1.38	1.30	1.17	1.15	1.26	1.33	1.30	1.23	1.20	1.12	1.12	1.19	1.27	1.25
	**Mean ± SD**	**1.45 ± 0.73**	**1.39 ± 0.67**	**1.27 ± 0.61**	**1.14 ± 0.55**	**1.00 ± 0.50**	**1.41 ± 0.76**	**1.45 ± 0.68**	**1.33 ± 0.61**	**1.20 ± 0.57**	**1.07 ± 0.55**	**1.21 ± 0.71**	**1.33 ± 0.72**	**1.30 ± 0.63**	**1.19 ± 0.57**	**1.07 ± 0.60**

Average maximal inspiratory displacement (mm) of 15 grid points located within genioglossus for 20 subjects. A – T denotes the 20 subjects. Data are expressed as mean ± SD.
