# Supplementary material for: Influence of respiratory mechanics and drive on genioglossus movement under ultrasound imaging
Source: PLoS One. 2018 Apr 16;13(4):e0195884. doi: 10.1371/journal.pone.0195884 (PMC5901985; doi:10.1371/journal.pone.0195884)
Supplement: S2 Table — (PDF) [file pone.0195884.s002.pdf]

Supporting Table 2. Mean maximal inspiratory displacement of 15 grid points during inspiratory resistive load experiment for 20 subjects.

	Point	1	2	3	4	5	6	7	8	9	10	11	12	13	14	15
**Spontaneous tidal breathing**	**A**	0.90	0.96	0.61	0.64	0.49	0.85	1.02	0.96	0.80	0.44	0.95	1.19	1.08	0.65	0.51
	**B**	0.46	0.70	0.76	0.69	0.49	0.44	0.59	0.69	0.49	0.63	0.86	0.80	0.94	0.87	0.63
	**C**	1.08	1.14	1.12	1.05	0.87	1.15	1.23	1.24	1.03	1.02	1.39	1.42	1.36	1.13	1.04
	**D**	1.11	1.23	0.99	0.92	0.93	1.12	1.24	1.26	1.24	1.24	0.60	0.93	1.22	1.22	0.98
	**E**	0.65	0.80	0.68	0.72	0.54	0.59	0.63	0.64	0.59	0.85	0.42	0.54	0.56	0.47	0.47
	**F**	1.23	0.94	0.67	0.63	0.76	1.45	1.33	1.27	0.98	0.73	1.08	1.51	1.27	0.91	0.60
	**G**	0.91	0.87	0.78	0.80	0.65	1.08	1.14	0.96	1.02	0.92	0.88	1.33	1.35	1.38	1.26
	**H**	0.83	0.80	0.70	0.76	0.83	0.93	0.91	0.83	0.95	0.88	1.03	1.06	1.10	1.07	0.98
	**I**	1.39	1.18	0.92	0.84	0.68	1.56	1.69	1.49	1.25	0.78	1.31	1.74	1.69	1.10	0.49
	**J**	0.99	0.79	0.65	0.40	0.36	0.78	0.87	0.58	0.47	0.33	0.69	0.83	0.58	0.57	0.59
	**K**	0.56	0.78	0.92	0.92	0.70	0.57	0.83	0.89	0.68	0.48	0.46	0.60	0.58	0.50	0.37
	**L**	1.00	1.14	1.08	0.79	0.46	1.30	1.40	1.27	1.12	1.07	1.18	1.53	1.72	1.53	1.32
	**M**	0.61	0.76	0.63	0.47	0.50	0.39	0.75	0.99	0.68	1.06	0.51	0.62	0.40	0.26	0.99
	**N**	2.10	2.94	2.64	1.89	1.34	2.61	3.20	2.94	2.37	1.87	2.16	3.23	2.96	1.89	1.28
	**O**	0.99	1.11	1.06	0.93	0.79	0.82	1.04	1.04	1.05	1.03	0.96	1.04	0.97	1.34	1.34
	**P**	0.72	0.72	0.64	0.66	0.61	0.70	0.87	0.98	0.95	0.87	0.61	1.13	1.24	1.17	1.06
	**Q**	0.88	0.79	0.76	0.64	0.52	0.90	0.99	0.74	0.57	0.50	0.92	0.89	0.69	0.38	0.22
	**R**	1.51	1.15	0.92	0.70	0.54	1.17	0.81	0.73	0.68	0.40	0.66	0.85	0.67	0.64	0.32
	**S**	0.21	0.24	0.29	0.31	0.28	0.30	0.34	0.39	0.41	0.26	0.29	0.33	0.32	0.22	0.21
	**T**	0.43	0.49	0.44	0.27	0.26	0.43	0.57	0.31	0.46	0.39	0.18	0.23	0.31	0.43	0.26
	**Mean ± SD**	**0.93 ± 0.43**	**0.98 ± 0.52**	**0.86 ± 0.47**	**0.75 ± 0.34**	**0.63 ± 0.25**	**0.96 ± 0.53**	**1.07 ± 0.59**	**1.01 ± 0.55**	**0.89 ± 0.44**	**0.79 ± 0.38**	**0.86 ± 0.45**	**1.09 ± 0.65**	**1.05 ± 0.62**	**0.89 ± 0.46**	**0.75 ± 0.39**
**Load A**	**A**	0.58	0.55	0.53	0.42	0.41	0.48	0.57	0.80	0.62	0.56	0.63	0.65	0.48	0.63	0.45
	**B**	0.34	0.34	0.38	0.44	0.36	0.44	0.36	0.36	0.46	0.37	0.36	0.44	0.42	0.45	0.38
	**C**	0.66	0.73	0.56	0.58	0.74	0.66	0.78	0.82	0.67	0.83	1.00	0.85	1.04	1.11	0.71
	**D**	0.75	0.72	0.39	0.55	0.76	0.56	0.49	0.61	0.68	0.76	0.67	0.73	0.88	0.67	0.73
	**E**	0.50	0.69	0.86	0.96	0.90	0.36	0.42	0.49	0.60	0.57	0.33	0.50	0.34	0.43	0.26
	**F**	0.96	0.88	0.97	0.77	0.61	1.40	1.25	1.12	1.05	0.82	1.55	1.80	1.83	1.84	2.10
	**G**	0.88	0.78	0.52	0.59	0.27	0.74	0.76	0.58	0.51	0.50	0.94	1.05	1.17	0.90	0.59
	**H**	0.34	0.35	0.45	0.33	0.43	0.63	0.46	0.40	0.45	0.47	0.49	0.55	0.60	0.52	0.31
	**I**	1.47	1.45	1.10	1.04	0.95	1.04	1.51	1.52	1.13	0.77	0.82	1.48	1.60	1.24	0.96
	**J**	0.88	0.82	0.60	0.60	0.42	0.93	0.93	0.71	0.63	0.44	1.10	1.03	0.86	0.70	0.69
	**K**	1.80	1.75	1.66	1.55	1.32	1.50	1.40	1.33	1.32	1.69	0.82	1.27	1.61	1.71	1.70
	**L**	0.70	0.74	0.68	0.62	0.44	0.74	0.98	0.74	0.86	0.70	0.81	1.14	1.12	1.17	0.77
	**M**	0.39	0.33	0.22	0.20	0.18	0.38	0.45	0.40	0.28	0.30	0.12	0.38	0.43	0.46	0.24
	**N**	1.72	2.53	2.13	1.52	1.16	1.30	2.20	2.03	1.64	1.34	0.74	1.68	1.62	1.36	0.92
	**O**	0.81	0.85	0.73	0.77	0.72	0.81	0.74	0.82	0.78	0.90	0.88	0.91	1.25	1.25	0.90
	**P**	0.70	0.76	0.82	0.84	0.83	0.71	0.81	0.83	0.94	0.96	0.73	0.89	0.98	1.05	1.08
	**Q**	0.55	0.52	0.42	0.41	0.46	0.48	0.65	0.70	0.70	0.70	0.51	0.67	0.45	0.49	0.37
	**R**	2.44	2.01	1.72	1.30	0.87	1.91	1.79	1.50	0.84	0.59	1.02	1.07	1.04	0.72	0.39
	**S**	0.59	0.61	0.73	0.74	0.65	0.39	0.64	0.67	0.70	0.70	0.43	0.43	0.46	0.61	0.62
	**T**	0.22	0.28	0.35	0.44	0.40	0.24	0.38	0.36	0.39	0.34	0.21	0.42	0.49	0.38	0.26
	**Mean ± SD**	**0.86 ± 0.57**	**0.88 ± 0.60**	**0.79 ± 0.51**	**0.73 ± 0.38**	**0.64 ± 0.30**	**0.79 ± 0.44**	**0.88 ± 0.51**	**0.84 ± 0.45**	**0.76 ± 0.33**	**0.72 ± 0.34**	**0.71 ± 0.34**	**0.90 ± 0.42**	**0.93 ± 0.47**	**0.88 ± 0.44**	**0.72 ± 0.48**
**Load B**	**A**	1.13	1.23	1.23	0.79	0.82	1.55	1.50	1.45	1.38	1.12	1.73	1.44	1.70	1.45	0.95
	**B**	1.42	1.23	1.27	1.65	1.40	1.26	1.39	1.26	0.98	1.07	1.46	1.32	1.12	1.12	0.90
	**C**	0.67	0.88	0.53	0.85	0.76	0.72	0.83	0.59	0.79	0.77	0.82	0.79	0.88	0.99	0.63
	**D**	0.78	0.82	0.91	1.01	0.95	0.70	0.78	0.92	0.89	0.45	1.11	1.13	1.13	0.59	0.38
	**E**	0.43	0.40	0.46	0.50	0.67	0.54	0.33	0.17	0.31	0.29	0.92	0.56	0.75	0.56	1.02
	**F**	1.39	1.12	1.00	0.70	0.63	1.65	1.39	1.61	0.97	0.83	1.68	1.88	2.02	1.97	1.01
	**G**	0.60	0.46	0.24	0.07	0.05	0.48	0.40	0.43	0.18	0.23	0.64	0.59	0.59	0.52	0.34
	**H**	0.50	0.43	0.49	0.46	0.52	0.48	0.50	0.51	0.56	0.61	0.43	0.48	0.62	0.67	0.55
	**I**	0.54	0.48	0.45	0.41	0.43	0.56	0.64	0.56	0.53	0.48	0.33	0.54	0.53	0.68	0.57
	**J**	1.00	0.82	0.74	0.68	0.72	0.92	0.86	0.84	0.73	0.79	0.78	0.94	0.82	0.81	0.82
	**K**	1.04	1.06	0.92	0.86	0.85	1.02	1.18	0.98	1.06	1.04	0.93	1.14	1.13	1.11	0.91
	**L**	0.94	0.83	0.75	0.72	0.49	1.00	1.13	0.94	0.79	0.72	1.28	1.31	1.13	1.02	0.79
	**M**	0.71	0.76	0.78	0.70	0.48	0.60	0.69	0.66	0.47	0.46	0.65	0.68	0.47	0.28	0.48

	**N**	1.92	3.01	2.59	2.31	1.95	1.62	3.23	3.33	3.06	2.37	1.83	2.88	3.04	2.64	2.19
	**O**	1.48	1.56	1.49	1.18	1.17	1.82	2.01	1.64	1.47	1.53	1.56	1.72	1.93	1.86	1.95
	**P**	0.28	0.30	0.31	0.46	0.46	0.42	0.39	0.39	0.37	0.41	0.47	0.44	0.40	0.37	0.46
	**Q**	0.51	0.54	0.37	0.20	0.12	0.46	0.75	0.54	0.35	0.16	0.39	0.59	0.43	0.32	0.32
	**R**	1.14	0.85	0.69	0.46	0.49	0.93	0.71	0.59	0.47	0.55	0.75	0.66	0.58	0.47	0.51
	**S**	0.20	0.18	0.20	0.31	0.19	0.65	0.63	0.56	0.31	0.23	0.44	0.12	0.22	0.19	0.16
	**T**	0.21	0.48	0.56	0.24	0.28	0.15	0.40	0.50	0.38	0.41	0.37	0.48	0.64	0.62	0.40
	**Mean ± SD**	**0.85 ± 0.47**	**0.87 ± 0.62**	**0.80 ± 0.55**	**0.73 ± 0.52**	**0.67 ± 0.45**	**0.88 ± 0.48**	**0.99 ± 0.69**	**0.92 ± 0.70**	**0.80 ± 0.64**	**0.73 ± 0.52**	**0.93 ± 0.50**	**0.99 ± 0.65**	**1.01 ± 0.69**	**0.91 ± 0.64**	**0.77 ± 0.51**
**Load C**	**A**	1.30	1.53	1.26	1.19	1.24	1.46	1.76	1.77	1.63	1.54	1.30	1.74	1.97	1.80	1.44
	**B**	0.57	0.91	0.63	0.56	0.52	0.55	0.55	0.70	0.74	0.46	0.60	0.52	0.59	0.57	0.43
	**C**	1.24	1.19	1.03	1.25	1.14	1.41	1.60	1.50	1.37	1.40	1.42	1.46	1.41	1.36	1.25
	**D**	0.98	1.09	1.02	0.85	0.64	0.98	0.97	0.88	0.91	1.27	1.04	1.73	1.22	1.21	1.51
	**E**	1.08	1.16	1.17	1.24	1.49	0.92	1.20	0.98	1.70	1.32	0.98	1.09	0.77	1.07	1.36
	**F**	0.86	0.78	0.76	0.60	0.58	1.02	0.95	0.92	0.84	0.76	1.20	1.13	1.05	0.91	0.82
	**G**	1.87	1.78	1.55	1.26	0.85	2.30	2.00	1.76	1.52	1.19	2.35	2.19	1.92	1.65	1.54
	**H**	1.06	0.85	0.73	0.65	0.72	0.77	1.15	1.05	0.85	0.73	0.81	0.95	0.99	0.99	0.88
	**I**	0.12	0.22	0.16	0.13	0.19	0.12	0.08	0.08	0.08	0.10	0.18	0.05	0.04	0.10	0.09
	**J**	2.51	1.61	1.91	1.85	2.02	2.95	2.36	1.86	2.33	2.65	2.69	2.52	2.20	2.32	2.18
	**K**	1.21	1.21	1.53	1.42	1.28	1.28	1.47	1.62	1.62	1.49	1.09	1.74	2.08	1.94	1.70
	**L**	1.71	1.61	1.50	1.39	1.46	2.26	2.16	1.99	1.73	1.34	2.66	2.48	2.12	1.73	1.22
	**M**	0.74	0.60	0.54	0.43	0.31	0.84	0.79	0.61	0.55	0.47	0.69	0.86	0.78	0.78	0.73
	**N**	0.67	0.97	1.07	1.01	0.78	0.92	1.21	1.10	1.09	0.89	1.19	1.26	1.22	1.25	0.95
	**O**	1.99	1.67	1.47	1.04	1.27	1.39	1.86	2.17	2.08	1.92	1.33	1.59	1.93	1.96	1.77
	**P**	0.62	0.70	0.69	0.73	0.73	0.79	0.79	0.93	0.90	0.90	0.97	0.94	1.00	0.95	0.99
	**Q**	1.22	1.17	0.95	1.08	1.02	1.27	1.30	1.16	1.16	1.13	1.56	1.50	1.40	1.38	1.40
	**R**	1.12	1.13	0.90	0.83	0.62	1.10	1.25	0.87	0.75	0.93	0.99	0.96	0.65	0.40	0.50
	**S**	0.68	0.62	0.43	0.42	0.41	0.76	0.81	0.80	0.91	0.87	0.23	0.40	0.41	0.59	0.65
	**T**	2.55	2.27	2.60	2.37	2.37	2.63	2.60	2.18	1.75	2.21	2.42	2.68	2.28	3.70	1.27
	**Mean ± SD**	**1.21 ± 0.64**	**1.15 ± 0.49**	**1.10 ± 0.56**	**1.01 ± 0.52**	**0.98 ± 0.56**	**1.29 ± 0.73**	**1.34 ± 0.64**	**1.25 ± 0.58**	**1.23 ± 0.56**	**1.18 ± 0.61**	**1.29 ± 0.73**	**1.39 ± 0.72**	**1.30 ± 0.67**	**1.33 ± 0.80**	**1.13 ± 0.51**

Average maximal inspiratory displacement (mm) of 15 grid points located within genioglossus for 20 subjects. A – T denotes the 20 subjects. Load A, B and C added an inspiratory resistance of 11.6 $cmH_2O/L/s$, 22.3 $cmH_2O/L/s$, and 75.2 $cmH_2O/L/s$ respectively. Data are expressed as mean ± SD.
