# Supplementary material for: Influence of respiratory mechanics and drive on genioglossus movement under ultrasound imaging
Source: PLoS One. 2018 Apr 16;13(4):e0195884. doi: 10.1371/journal.pone.0195884 (PMC5901985; doi:10.1371/journal.pone.0195884)
Supplement: S3 Table — (PDF) [file pone.0195884.s003.pdf]

**Supporting Table 3. Mean maximal inspiratory displacement of 15 grid points during lung volume alteration experiment for 20 subjects.**

| | Point | 1 | 2 | 3 | 4 | 5 | 6 | 7 | 8 | 9 | 10 | 11 | 12 | 13 | 14 | 15 |
|---|---|---|---|---|---|---|---|---|---|---|---|---|---|---|---|---|
| **Negative extrathoracic pressure** | **A** | 0.55 | 0.53 | 0.48 | 0.44 | 0.34 | 0.71 | 0.64 | 0.77 | 0.71 | 0.59 | 0.69 | 0.70 | 0.78 | 0.80 | 0.69 |
| | **B** | 0.06 | 0.05 | 0.04 | 0.13 | 0.13 | 0.29 | 0.19 | 0.08 | 0.20 | 0.16 | 0.46 | 0.30 | 0.40 | 0.05 | 0.20 |
| | **C** | 0.13 | 0.19 | 0.10 | 0.07 | 0.14 | 0.21 | 0.21 | 0.18 | 0.06 | 0.12 | 0.12 | 0.17 | 0.17 | 0.09 | 0.08 |
| | **D** | 0.16 | 0.19 | 0.05 | 0.06 | 0.13 | 0.14 | 0.14 | 0.22 | 0.20 | 0.16 | 0.08 | 0.33 | 0.49 | 0.32 | 0.31 |
| | **E** | 0.33 | 0.32 | 0.44 | 0.32 | 0.24 | 0.36 | 0.30 | 0.43 | 0.31 | 0.48 | 0.38 | 0.42 | 0.48 | 0.54 | 0.35 |
| | **F** | 2.04 | 1.80 | 1.02 | 0.64 | 0.58 | 1.23 | 1.02 | 0.65 | 0.49 | 0.42 | 1.18 | 1.22 | 0.89 | 0.59 | 0.37 |
| | **G** | 0.21 | 0.18 | 0.10 | 0.31 | 0.14 | 0.23 | 0.11 | 0.15 | 0.19 | 0.11 | 0.20 | 0.23 | 0.25 | 0.33 | 0.15 |
| | **H** | 0.11 | 0.22 | 0.29 | 0.24 | 0.15 | 0.22 | 0.07 | 0.19 | 0.24 | 0.17 | 0.27 | 0.23 | 0.16 | 0.26 | 0.27 |
| | **I** | 0.51 | 0.73 | 0.81 | 1.67 | 1.82 | 0.90 | 0.93 | 0.68 | 0.50 | 0.27 | 1.07 | 1.49 | 1.78 | 1.51 | 0.60 |
| | **J** | 0.11 | 0.00 | 0.05 | 0.07 | 0.20 | 0.09 | 0.04 | 0.04 | 0.09 | 0.11 | 0.10 | 0.10 | 0.14 | 0.03 | 0.07 |
| | **K** | 0.61 | 0.35 | 0.08 | 0.25 | 0.18 | 0.88 | 0.69 | 0.51 | 0.41 | 0.38 | 0.61 | 0.95 | 1.01 | 0.57 | 0.20 |
| | **L** | 0.19 | 0.19 | 0.26 | 0.24 | 0.14 | 0.09 | 0.39 | 0.22 | 0.28 | 0.15 | 0.02 | 0.42 | 0.40 | 0.28 | 0.16 |
| | **M** | 0.43 | 0.09 | 0.08 | 0.17 | 0.07 | 0.45 | 0.46 | 0.30 | 0.42 | 0.18 | 0.94 | 0.69 | 0.39 | 0.44 | 0.53 |
| | **N** | 0.16 | 0.11 | 0.17 | 0.44 | 0.18 | 0.10 | 0.08 | 0.45 | 0.29 | 0.20 | 0.63 | 0.45 | 0.56 | 0.48 | 0.17 |
| | **O** | 0.45 | 0.52 | 0.54 | 0.34 | 0.15 | 0.46 | 0.50 | 0.41 | 0.52 | 0.37 | 0.26 | 0.45 | 0.63 | 0.65 | 0.15 |
| | **P** | 0.06 | 0.08 | 0.04 | 0.02 | 0.27 | 0.63 | 0.34 | 0.17 | 0.32 | 0.34 | 0.46 | 0.53 | 0.30 | 0.24 | 0.05 |
| | **Q** | 0.22 | 0.34 | 0.22 | 0.23 | 0.26 | 0.39 | 0.42 | 0.41 | 0.42 | 0.30 | 0.73 | 0.74 | 0.61 | 0.47 | 0.24 |
| | **R** | 0.26 | 0.56 | 0.50 | 0.19 | 0.11 | 0.42 | 0.69 | 0.70 | 0.50 | 0.08 | 0.42 | 0.76 | 1.03 | 0.98 | 0.83 |
| | **S** | 0.13 | 0.11 | 0.10 | 0.08 | 0.07 | 0.13 | 0.12 | 0.10 | 0.09 | 0.08 | 0.09 | 0.09 | 0.09 | 0.10 | 0.11 |
| | **T** | 0.23 | 0.18 | 0.15 | 0.08 | 0.14 | 0.31 | 0.27 | 0.23 | 0.11 | 0.41 | 0.39 | 0.51 | 0.11 | 0.05 | 0.09 |
| | **Mean ± SD** | **0.35 ± 0.43** | **0.34 ± 0.39** | **0.28 ± 0.27** | **0.30 ± 0.56** | **0.27 ± 0.38** | **0.41 ± 0.31** | **0.38 ± 0.29** | **0.34 ± 0.22** | **0.32 ± 0.18** | **0.25 ± 0.15** | **0.46 ± 0.34** | **0.54 ± 0.37** | **0.53 ± 0.41** | **0.44 ± 0.36** | **0.28 ± 0.22** |
| **Spontaneous tidal breathing** | **A** | 0.47 | 0.55 | 0.46 | 0.39 | 0.31 | 0.61 | 0.43 | 0.41 | 0.40 | 0.24 | 0.77 | 0.73 | 0.59 | 0.42 | 0.33 |
| | **B** | 0.20 | 0.11 | 0.31 | 0.11 | 0.13 | 0.29 | 0.25 | 0.11 | 0.11 | 0.13 | 0.22 | 0.15 | 0.15 | 0.13 | 0.21 |
| | **C** | 0.24 | 0.17 | 0.22 | 0.28 | 0.28 | 0.21 | 0.28 | 0.25 | 0.37 | 0.33 | 0.16 | 0.28 | 0.30 | 0.35 | 0.36 |
| | **D** | 0.45 | 0.58 | 0.48 | 0.30 | 0.23 | 0.53 | 0.46 | 0.50 | 0.44 | 0.30 | 0.41 | 0.73 | 0.62 | 0.48 | 0.25 |
| | **E** | 0.67 | 0.72 | 0.71 | 0.71 | 0.62 | 0.50 | 0.72 | 0.84 | 0.83 | 0.51 | 0.71 | 0.85 | 0.89 | 0.61 | 0.26 |
| | **F** | 0.87 | 0.63 | 0.50 | 0.50 | 0.37 | 1.20 | 1.05 | 0.61 | 0.55 | 0.69 | 1.19 | 1.43 | 1.10 | 0.47 | 0.26 |
| | **G** | 0.62 | 0.55 | 0.30 | 0.32 | 0.33 | 1.24 | 1.26 | 1.05 | 0.87 | 0.51 | 1.36 | 1.92 | 1.66 | 0.97 | 0.83 |
| | **H** | 0.46 | 0.35 | 0.40 | 0.22 | 0.18 | 0.48 | 0.43 | 0.41 | 0.39 | 0.36 | 0.45 | 0.58 | 0.48 | 0.48 | 0.55 |
| | **I** | 0.34 | 0.34 | 0.38 | 0.45 | 0.40 | 0.49 | 0.50 | 0.42 | 0.40 | 0.33 | 0.72 | 0.87 | 0.65 | 0.54 | 0.44 |
| | **J** | 0.12 | 0.09 | 0.14 | 0.11 | 0.08 | 0.11 | 0.10 | 0.09 | 0.03 | 0.11 | 0.11 | 0.23 | 0.19 | 0.20 | 0.28 |
| | **K** | 0.67 | 0.72 | 0.57 | 0.35 | 0.28 | 0.59 | 0.57 | 0.58 | 0.55 | 0.40 | 0.55 | 0.72 | 0.94 | 0.84 | 0.45 |
| | **L** | 0.22 | 0.20 | 0.30 | 0.21 | 0.23 | 0.22 | 0.32 | 0.30 | 0.36 | 0.33 | 0.29 | 0.18 | 0.28 | 0.33 | 0.26 |
| | **M** | 1.02 | 0.84 | 0.61 | 0.43 | 0.28 | 0.89 | 0.85 | 0.79 | 0.24 | 0.13 | 0.86 | 0.92 | 0.52 | 0.34 | 0.38 |
| | **N** | 0.31 | 0.59 | 0.36 | 0.36 | 0.47 | 0.50 | 0.35 | 0.57 | 0.42 | 0.40 | 0.48 | 0.69 | 0.66 | 0.44 | 0.35 |
| | **O** | 0.22 | 0.17 | 0.14 | 0.24 | 0.26 | 0.36 | 0.31 | 0.21 | 0.14 | 0.10 | 0.24 | 0.47 | 0.49 | 0.39 | 0.25 |
| | **P** | 0.52 | 0.40 | 0.29 | 0.13 | 0.12 | 0.52 | 0.57 | 0.54 | 0.41 | 0.33 | 0.55 | 0.70 | 0.66 | 0.51 | 0.47 |
| | **Q** | 0.25 | 0.23 | 0.16 | 0.21 | 0.16 | 0.24 | 0.19 | 0.18 | 0.20 | 0.11 | 0.40 | 0.48 | 0.32 | 0.08 | 0.19 |
| | **R** | 0.24 | 0.24 | 0.17 | 0.18 | 0.12 | 0.23 | 0.35 | 0.29 | 0.39 | 0.28 | 0.45 | 0.62 | 0.59 | 0.46 | 0.38 |
| | **S** | 0.55 | 0.52 | 0.49 | 0.47 | 0.48 | 0.60 | 0.60 | 0.62 | 0.62 | 0.63 | 0.56 | 0.68 | 0.75 | 0.78 | 0.75 |
| | **T** | 0.45 | 0.34 | 0.34 | 0.34 | 0.15 | 0.67 | 0.57 | 0.33 | 0.17 | 0.24 | 0.65 | 0.68 | 0.39 | 0.24 | 0.23 |
| | **Mean ± SD** | **0.44 ± 0.24** | **0.42 ± 0.22** | **0.37 ± 0.16** | **0.32 ± 0.15** | **0.27 ± 0.14** | **0.52 ± 0.30** | **0.51 ± 0.29** | **0.45 ± 0.25** | **0.39 ± 0.22** | **0.32 ± 0.17** | **0.56 ± 0.32** | **0.70 ± 0.41** | **0.61 ± 0.35** | **0.45 ± 0.22** | **0.37 ± 0.17** |
| **Positive extrathoracic** | **A** | 0.72 | 0.49 | 0.15 | 0.28 | 0.38 | 1.09 | 0.68 | 0.29 | 0.18 | 0.50 | 0.95 | 1.12 | 0.64 | 0.18 | 0.17 |
| | **B** | 0.11 | 0.19 | 0.12 | 0.08 | 0.08 | 0.26 | 0.24 | 0.31 | 0.10 | 0.09 | 0.18 | 0.16 | 0.12 | 0.25 | 0.04 |
| | **C** | 0.34 | 0.05 | 0.14 | 0.32 | 0.24 | 0.17 | 0.06 | 0.15 | 0.12 | 0.16 | 0.18 | 0.28 | 0.15 | 0.03 | 0.08 |
| | **D** | 0.75 | 0.65 | 0.44 | 0.51 | 0.50 | 0.67 | 0.87 | 0.67 | 0.44 | 0.35 | 1.00 | 1.07 | 0.85 | 0.78 | 0.32 |
| | **E** | 0.58 | 0.63 | 0.45 | 0.55 | 0.45 | 0.67 | 0.49 | 0.46 | 0.39 | 0.34 | 0.48 | 0.41 | 0.34 | 0.41 | 0.38 |
| | **F** | 1.06 | 0.85 | 0.61 | 0.51 | 0.72 | 1.18 | 0.98 | 0.52 | 0.34 | 0.22 | 1.14 | 1.20 | 0.62 | 0.23 | 0.26 |
| | **G** | 0.83 | 0.97 | 0.79 | 0.65 | 0.50 | 1.86 | 1.52 | 1.24 | 1.24 | 0.76 | 1.59 | 2.36 | 2.07 | 1.05 | 0.95 |
| | **H** | 0.48 | 0.50 | 0.38 | 0.43 | 0.65 | 0.53 | 0.44 | 0.50 | 0.53 | 0.57 | 0.89 | 0.88 | 0.82 | 0.76 | 0.83 |
| | **I** | 0.19 | 0.19 | 0.50 | 0.53 | 0.42 | 0.30 | 0.31 | 0.34 | 0.35 | 0.33 | 0.56 | 0.54 | 0.45 | 0.45 | 0.40 |
| | **J** | 0.15 | 0.32 | 0.58 | 0.61 | 0.18 | 0.41 | 0.28 | 0.23 | 0.18 | 0.28 | 0.60 | 0.73 | 0.68 | 0.38 | 0.30 |
| | **K** | 0.77 | 0.82 | 0.75 | 0.56 | 0.26 | 0.74 | 0.47 | 0.21 | 0.13 | 0.13 | 0.58 | 0.43 | 0.23 | 0.25 | 0.32 |
| | **L** | 0.03 | 0.05 | 0.14 | 0.02 | 0.11 | 0.05 | 0.10 | 0.09 | 0.09 | 0.24 | 0.08 | 0.05 | 0.06 | 0.08 | 0.05 |
| | **M** | 1.34 | 1.05 | 0.82 | 0.67 | 0.34 | 1.30 | 1.33 | 0.91 | 0.69 | 0.21 | 1.13 | 1.57 | 1.07 | 0.71 | 0.40 |

| | | | | | | | | | | | | | | | | |
|---|---|---|---|---|---|---|---|---|---|---|---|---|---|---|---|---|
| | **N** | 0.55 | 0.55 | 0.17 | 0.12 | 0.43 | 0.58 | 0.75 | 0.55 | 0.42 | 0.35 | 0.49 | 0.82 | 0.26 | 0.22 | 0.11 |
| | **O** | 0.18 | 0.17 | 0.27 | 0.12 | 0.11 | 0.25 | 0.24 | 0.23 | 0.19 | 0.11 | 0.25 | 0.38 | 0.37 | 0.25 | 0.09 |
| | **P** | 0.30 | 0.12 | 0.02 | 0.11 | 0.07 | 0.33 | 0.57 | 0.54 | 0.29 | 0.14 | 0.62 | 0.69 | 0.59 | 0.21 | 0.14 |
| | **Q** | 0.85 | 0.56 | 0.53 | 0.30 | 0.07 | 0.82 | 0.83 | 0.68 | 0.46 | 0.43 | 0.73 | 0.60 | 0.34 | 0.19 | 0.30 |
| | **R** | 0.84 | 0.66 | 0.51 | 0.38 | 0.27 | 0.36 | 0.89 | 0.56 | 0.62 | 0.47 | 0.45 | 0.95 | 1.33 | 1.24 | 0.72 |
| | **S** | 0.47 | 0.45 | 0.39 | 0.24 | 0.31 | 0.55 | 0.56 | 0.56 | 0.56 | 0.56 | 0.58 | 0.68 | 0.76 | 0.75 | 0.72 |
| | **T** | 1.28 | 0.61 | 0.64 | 0.23 | 0.19 | 1.90 | 1.58 | 1.13 | 0.08 | 0.72 | 0.83 | 1.44 | 1.62 | 1.21 | 0.07 |
| | **Mean ± SD** | **0.59 ± 0.38** | **0.49 ± 0.30** | **0.42 ± 0.24** | **0.36 ± 0.21** | **0.31 ± 0.19** | **0.70 ± 0.52** | **0.66 ± 0.44** | **0.51 ± 0.31** | **0.37 ± 0.28** | **0.35 ± 0.20** | **0.67 ± 0.38** | **0.82 ± 0.54** | **0.67 ± 0.52** | **0.48 ± 0.37** | **0.33 ± 0.27** |

Average maximal inspiratory displacement (mm) of 15 grid points located within genioglossus for 20 subjects. A – T denotes the 20 subjects. Extrathoracic negative pressure increased end-expiratory lung volume (EELV) by ~1000 mL and extrathoracic positive pressure reduced EELV by ~500 mL respectively. Data are expressed as mean ± SD.
