# Supplementary material for: Influence of respiratory mechanics and drive on genioglossus movement under ultrasound imaging
Source: PLoS One. 2018 Apr 16;13(4):e0195884. doi: 10.1371/journal.pone.0195884 (PMC5901985; doi:10.1371/journal.pone.0195884)
Supplement: S4 Table — (PDF) [file pone.0195884.s004.pdf]

Supporting Table 4. Respiratory variables for the lung volume alteration experiment.

	Spontaneous tidal breathing	Negative extra-thoracic pressure	Positive extra-thoracic pressure	Outside chamber tidal breathing	Inside chamber tidal breathing
Number of subjects (n)	20	20	20	6	6
Tidal volume (mL)	375 ± 163	374 ± 135	365 ± 235	416 ± 134	439 ± 166
Respiratory rate (breaths / min)	15.4 ± 2.7	16.5 ± 3.2	17.4 ± 3.7	13.3 ± 1.9	13.8 ± 1.8
Respiratory cycle length (sec)	4.10 ± 0.81	3.81 ± 1.02	3.62 ± 0.86	4.58 ± 0.72	4.43 ± 0.60
Inspiratory time (sec)	1.26 ± 0.31	1.13 ± 0.40	1.13 ± 0.27	1.19 ± 0.31	0.97 ± 0.41

Data are expressed as mean ± SD.
